# Late-life physical activity, midlife-to-late-life activity patterns, *APOE* ε4 genotype, and cognitive impairment among Chinese older adults: a population-based observational study

**DOI:** 10.1186/s12966-024-01691-7

**Published:** 2025-01-09

**Authors:** Xunying Zhao, Xueyao Wu, Tianpei Ma, Jinyu Xiao, Xin Chen, Mingshuang Tang, Li Zhang, Tao Zhang, Mengyu Fan, Jiaqiang Liao, Ben Zhang, Xia Jiang, Jiayuan Li

**Affiliations:** 1 Department of Epidemiology and Health Statistics and West China Institute of Preventive and Medical Integration for Major Diseases, West China School of Public Health and West China Fourth Hospital, Sichuan University, Chengdu, Sichuan China; 2 Department of Nutrition and Food Hygiene, West China School of Public Health and West China Fourth Hospital, Sichuan University, Chengdu, China; 3 Hainan General Hospital and Hainan Affiliated Hospital, Hainan Medical University, Haikou, China; 4 Department of Clinical Neuroscience, Center for Molecular Medicine, Karolinska Institutet, Solna, Stockholm, Sweden

**Keywords:** Physical activity, Cognitive impairment, Cognitive aging, *APOE* ε4 genotype, Older people

## Abstract

**Background:**

Although physical activity (PA) has been linked to cognitive health, the nuanced relationships between different dimensions of PA and cognitive impairment remain inconclusive. This study investigated associations between late-life PA levels, midlife-to-late-life activity patterns, and cognitive impairment in Chinese older adults, considering potential moderation by apolipoprotein E (*APOE*) ε4 genotype.

**Methods:**

We analyzed baseline data from 6,899 participants (median age 68 years, 55.78% female) in the West China Health and Aging Cohort study, with 6,575 participants having *APOE* genotyping data. Late-life PA and midlife-to-late-life activity patterns were assessed using the Global Physical Activity Questionnaire and a standardized question, respectively. Cognitive function was evaluated using the Chinese version of Mini-Mental State Examination. Logistic regression models were used to examine associations.

**Results:**

Compared to low PA level, moderate (odds ratio [OR] = 0.74, 95% confidence interval [CI] = 0.55 ~ 0.99) and high PA levels (OR = 0.60, 95%CI = 0.48 ~ 0.75) were associated with lower risk of cognitive impairment. Engaging in work-, transport-, recreation-related, and moderate-intensity PA were each significantly associated with lower cognitive impairment risk. Maintaining activity levels from midlife to late life was associated with lower cognitive impairment risk compared to decreasing levels (OR = 0.75, 95%CI = 0.60 ~ 0.94). These associations were more pronounced in *APOE* ε4 non-carriers, with an interaction observed between *APOE* ε4 genotype and recreation-related PA (*P*-value = 0.04).

**Conclusions:**

Our findings underscore the multifaceted benefits of PA in mitigating cognitive impairment risk among older Chinese adults. Public health strategies should focus on promoting overall late-life PA levels, especially moderate-intensity PA, and maintaining activity levels comparable to midlife, with potential for personalized interventions based on genetic risk profiles.

**Supplementary Information:**

The online version contains supplementary material available at 10.1186/s12966-024-01691-7.

## Background

The rapid progression of global aging has led to an exponential increase in the burden of cognitive impairment and dementia [1]. Given the current lack of effective treatments, identifying modifiable factors for cognitive decline prevention presents an opportunity to develop targeted strategies for promoting cognitive health and reducing the risk of dementia in older adults [2, 3]. Among these factors, regular physical activity (PA) has emerged as a key intervenable element [2, 4, 5]. Recent meta-analyses have demonstrated that moderate- and vigorous-intensity PA (MVPA) is beneficial for maintaining cognitive functions in older age [5], and overall PA levels are positively associated with better late-life cognition [6].

PA is a multifaceted behavior encompassing various dimensions such as type, intensity, and changing patterns [7]. Understanding its benefits requires considering comprehensive aspects beyond just overall PA levels. While most studies have focused on the effects of overall or recreation-related PA on cognitive function, evidence regarding other types (e.g., work- and transport-related) remains limited and inconsistent [8, 9]. Beyond activity types, determining optimal PA intensity for older adults is crucial, as age-related health decline may increase the difficulty and injury risks associated with vigorous-intensity PA (VPA) [10, 11]. However, the necessity of VPA for this population remains debatable [12, 13]. Additionally, PA levels typically declines with the aging process [10]. Although research has linked PA levels at various life stages to late-life cognitive function [5], few studies have examined the impact of maintaining or altering PA levels from midlife to late life.

Genetic factors, particularly the apolipoprotein E (*APOE*) ε4 allele, also play a crucial role in cognitive function and are well-established risk factor for cognitive decline and dementia [14, 15]. Modifiable lifestyle factors may influence *APOE* ε4 fragments levels, potentially mitigating cognitive decline risk [16–18]. While accumulating studies have evaluated the interaction between PA and *APOE* ε4 genotype on cognitive impairment risk, results remain contradictory, with some reporting evidence of *APOE*-PA interaction and others not [18–22]. Moreover, few studies have evaluated how various dimensions of PA might interact with *APOE* ε4 genotype to affect cognitive impairment risk.

Therefore, this study aimed to investigate the relationships between PA profiles, *APOE* ε4 genotype, and cognitive function in older adults, using data from 6,899 Chinese older adults from the West China Health and Aging Cohort (WCHAC) study. We evaluated associations between cognitive impairment risk and late-life PA levels (including overall, domain-specific, and intensity-specific PA), as well as midlife-to-late-life activity patterns. Additionally, we explored potential moderating effects of different PA dimensions on the relationship between *APOE* ε4 genotype and cognitive impairment.

## Methods

### Study design

The study adheres to the Strengthening the Reporting of Observational Studies in Epidemiology (STROBE) guidelines (https://www.strobe-statement.org/, Supplementary File 1). Participants were recruited from the WCHAC, approved by the Medical Ethics Committee of West China Fourth Hospital of Sichuan University (HXSY-EC-2022034) [23]. Since 2022, the cohort has been investigated in the service area of the Hongguang Community Health Service Center, Pidu District, Chengdu, utilizing a bidirectional cohort design. In this study, we utilized the baseline data of the prospective cohort collected from 2022 to 2023, including face-to-face questionnaire survey data, physical measurements, and genotype data (Supplementary File 2).

### Study participants

**Fig. 1:**
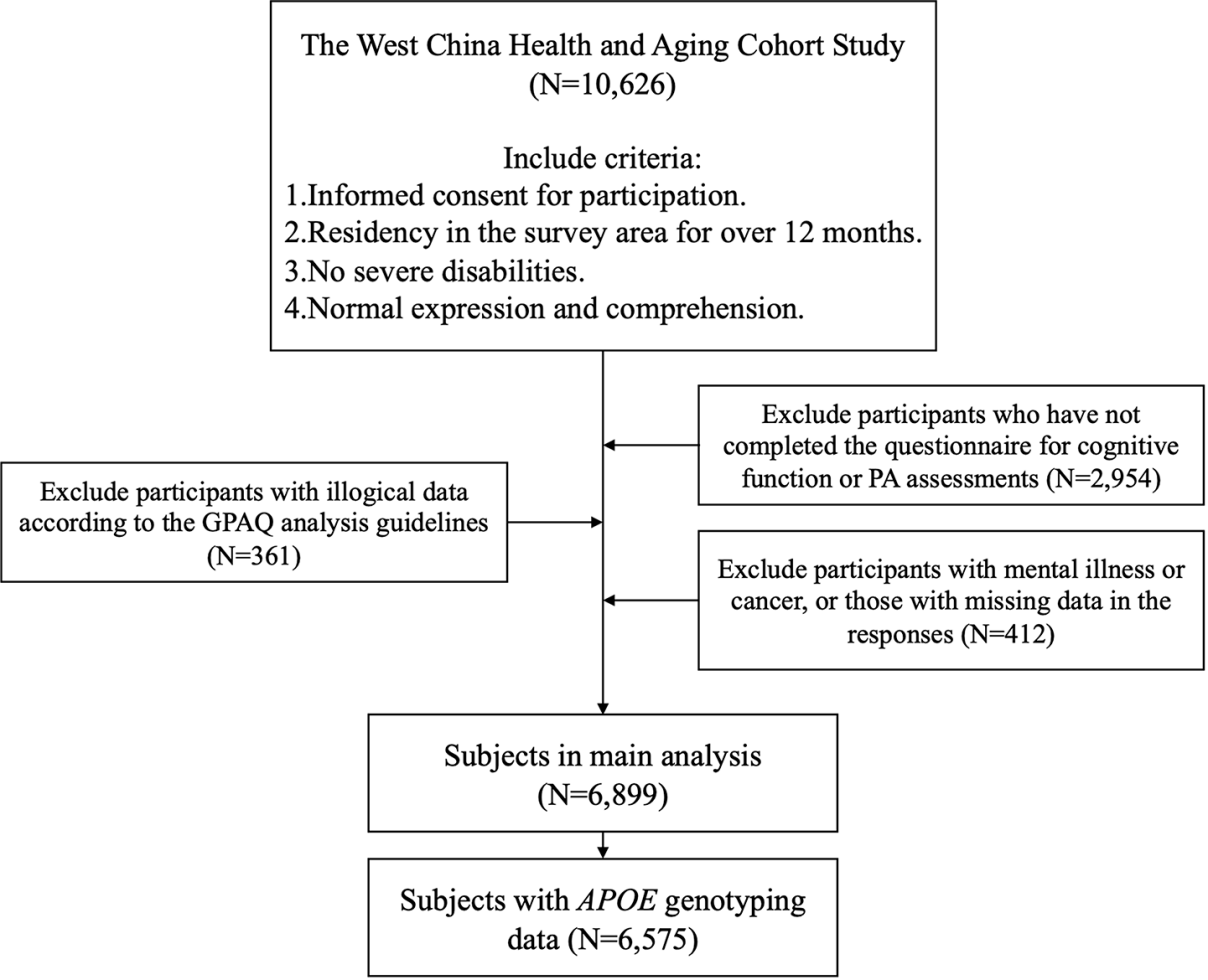
The flowchart outlining participant selection of the study. GPAQ (global physical activity questionnaire), PA (physical activity), *APOE* (apolipoprotein E)

At baseline, 10,626 participants aged around 60 or above were enrolled, satisfying the following inclusion criteria: (i) informed consent; (ii) residence in the survey area for ≥ 12 months; (iii) no severe disabilities; and (iv) normal abilities for expression and comprehension. After excluding 2,954 participants without cognitive function or PA assessments, 361 with illogical data of PA questionnaire, and 412 with mental illness, cancer, or missing responses regarding mental illness and cancer, 6,899 participants were included in the main analysis. Among these participants, 6,575 with *APOE* genotyping data were further included to analyses examining *APOE*-PA interactions and joint effects of PA levels and *APOE* ε4 genotype. The flowchart outlining participant selection is detailed in Fig. 1.

### Assessment of late-life physical activity

Self-reported PA in late life was assessed at recruitment using the Global Physical Activity Questionnaire version-2 (GPAQ-2). This tool collected information on intensity-specific PA in work (including housework), transport, and recreation during a typical week [24]. Following GPAQ guidelines, overall PA levels were calculated as the sum of metabolic equivalent tasks (METs) minutes per week of MVPA across activity types [25]. Moderate-intensity PA (MPA) was assigned 4.0 METs and VPA 8.0 METs. Participants were categorized into low (< 600 MET-minutes/week), moderate (≥ 600 and < 1200 MET-minutes/week), and high (≥ 1200 MET-minutes/week) PA groups based on World Health Organization (WHO) recommendations [25, 26]. We further defined domain-specific PA engagement based on participation in each activity type (work-, transport-, and recreation-related PA), and intensity-specific PA engagement based on participation in MPA or VPA.

### Assessment of midlife-to-late-life activity patterns

Midlife-to-late-life activity patterns were assessed using a 5-point scale question: “Compared to your current PA level, was your overall PA level (including all work-, transport-, and recreation-related activities) higher or lower when you were 40 ~ 60 years old?” Response included: “much higher” (1 point), “higher” (2 points), “unchanged” (3 points), “lower” (4 points), and “much lower” (5 points). We categorized responses as “decreasing activity levels” (1 ~ 2 points), “maintaining activity levels” (3 points), and “increasing activity levels” (4 ~ 5 points).

### Assessment of cognitive function

Cognitive function was assessed using the Chinese version of Mini-Mental State Examination (C-MMSE), which evaluates 5 domains including orientation, memory, attention, computation, and language abilities, with a total score of 30 points [27]. Higher C-MMSE scores indicate better cognitive performance. Dichotomized cognitive impairment was defined using validated education-based C-MMSE cut-off points: 16/17 for illiterate individuals, 19/20 for those with primary education, and 23/24 for those with junior and higher education [28].

### *APOE* genotyping

Fasting blood samples were collected in EDTA tubes by trained nurses. Genotyping was performed using Illumina Infinium Asian Screening Array Multiple Disease (Illumina-ASAMD), which contains ~ 740,000 markers. Quality control procedure was listed in Supplementary File 2. *APOE* genotyping was determined by two genetic variants - rs429358 and rs7412. Participants with ε2/ε4, ε3/ε4, and ε4/ε4 genotypes were grouped as *APOE* ε4 carriers, while those with ε2/ε2, ε2/ε3, and ε3/ε3 genotypes were grouped as *APOE* ε4 non-carriers [29].

### Covariates

A wide range of covariates were assessed at recruitment, encompassing sociodemographic, anthropometric, behavioral, and health status variables. Sociodemographic data included age, sex, education attainment, and major occupation before or after retirement. Anthropometric data included body mass index (BMI) and waist-to-hip ratio (WHR). Behavioral data included dietary habits, smoking status, drinking status, and sedentary time. Dietary habits over the past year were assessed by a qualitative food frequency questionnaire. Healthy dietary habit was defined as meeting at least 4 of the following 5 criteria: consuming fresh vegetables ≥ 7 days/week, fresh fruits ≥ 7 days/week, red meat 1–6 days/week, legumes ≥ 4 days/week, and fish ≥ 1 day/week [30]. Health status variables included functional limitations and self-reported histories of diagnosed hypertension or diabetes. Functional limitations were defined as the inability to independently perform any item of the activities of daily living (ADL) or instrumental activities of daily living (IADL). ADLs included dressing, bathing, feeding, transferring, toileting, and maintaining continence, while IADLs encompassed doing housework, cooking, shopping, managing finances, taking medication, and using the telephone [31, 32].

### Statistical analysis

Continuous variables were described with mean and standard deviation (or median and interquartile range [IQR]), and categorical variables with frequency and percentage. Differences between cognitive status groups were assessed using student’s *t*-test or *Wilcoxon* rank-sum test for continuous variables, and *Chi-square* test for categorical variables.

Logistic regression models were performed to assess the associations between each PA variable and risk of cognitive impairment. Categories of overall PA levels, domain- and intensity-specific PA engagement, and midlife-to-late-life activity patterns were evaluated as the independent variables separately. Crude models adjusted for age and sex, and multivariate-adjusted models adjusted, on top of crude model, for education attainment, major occupation before or after retirement, BMI, WHR, dietary habits, smoking status, drinking status, sedentary time, functional limitations, and histories of diagnosed hypertension and diabetes, to account for potential confounding in the PA-cognition relationship. Multivariate-adjusted models for domain- and intensity-specific PA and midlife-to-late-life activity patterns were additionally adjusted for overall PA levels.

To assess linear trends in overall late-life PA, we first conducted logistic regression models entering the median value of each overall PA level category as a continuous variable, and then conducted restricted cubic spline (RCS) regression models with continuous MET-minutes/week. We performed stratified analyses by age (< 68 vs. ≥68 years, based on median age) and sex to identify targeted subpopulations with the best public health implications. For midlife-to-late-life activity patterns, we further stratified by overall PA levels to examine the persistence of associations across different PA levels.

To investigate potential moderating effects of PA variables on the relationship between *APOE* ε4 genotype and late-life cognitive impairment, we first conducted a stratified analysis by *APOE* ε4 genotype category. We then tested for multiplicative interaction using the likelihood ratio test, comparing models with and without product terms of PA variables and *APOE* ε4 genotype [33]. Finally, we examined associations between combined PA variables and *APOE* ε4 genotype groups and cognitive impairment risk.

Several sensitivity analyses were conducted: (i) using continuous C-MMSE scores as outcomes; (ii) excluding participants with functional limitations; (iii) categorizing PA levels based on tertiles to ensure roughly equal group sizes; and (iv) repeating the main analyses in 6,575 participants with *APOE* genotyping data.

## Results

### Characteristics of the study participants

The demographic characteristics of our participants are listed in Table 1. The study involved 6,899 participants with a median age of 68 years (IQR 65, 73), of which 3,848 (55.78%) were females. Among 6,575 participants who were genotyped for *APOE*, 1103 (15.99%) were *APOE* ε4 carriers. Participants with cognitive impairment were more likely to be older, female, less educated, work in manual labor, have unhealthy dietary habits, smoke less, drink less, spend less time being sedentary, have functional limitations, and were more likely to be *APOE* ε4 carriers.

**Table 1:** Characteristics of the 6,899 participants by cognitive function

	Overall	Cognitive impairment	Cognitively normal	***P***-value
*N*	6899	1871	5028	
**Basic Characteristics**				
Age (years)	68 (65, 73)	69 (65, 74)	68 (65, 72)	< 0.01
Sex (female, %)	3848 (55.78%)	1198 (64.03%)	2650 (52.70%)	< 0.01
Education (%)				< 0.01
Illiterate	2307 (33.44%)	777 (41.53%)	1530 (30.43%)	
Primary	1519 (22.02%)	341 (18.23%)	1178 (23.43%)	
Junior and higher	3073 (44.54%)	753 (40.25%)	2320 (46.14%)	
Occupation (%)				< 0.01
Technical and managerial personnel	953 (13.81%)	129 (6.89%)	824 (16.39%)	
Service and sales personnel	1045 (15.15%)	215 (11.49%)	830 (16.51%)	
Manual worker	4887 (70.84%)	1524 (81.45%)	3363 (66.89%)	
Body mass index (kg/m^2^)	24.40 (22.40, 26.60)	24.30 (22.30, 26.70)	24.40 (22.40, 26.50)	0.80
Waist-to-hip ratio	0.89 (0.85, 0.93)	0.89 (0.85, 0.93)	0.89 (0.85, 0.93)	0.32
Healthy dietary habits (yes, %)	495 (7.17%)	103 (5.51%)	392 (7.80%)	< 0.01
Smoking (%)				< 0.01
Never	5149 (74.63%)	1483 (79.26%)	3666 (72.91%)	
Former	744 (10.78%)	160 (8.55%)	584 (11.61%)	
Current	1006 (14.58%)	228 (12.19%)	778 (15.47%)	
Alcohol intake (%)				< 0.01
Hardly ever drinks	4585 (66.46%)	1372 (73.33%)	3213 (63.90%)	
Less than weekly	1344 (19.48%)	273 (14.59%)	1071 (21.30%)	
Weekly	970 (14.06%)	226 (12.08%)	744 (14.80%)	
Sedentary time (minutes/day)	240 (120, 330)	180 (120, 300)	240 (150, 360)	< 0.01
Functional limitations (yes, %)	293 (4.25%)	145 (7.75%)	148 (2.94%)	< 0.01
Diabetes (yes, %)	1079 (15.64%)	302 (16.14%)	777 (15.45%)	0.51
Hypertension (yes, %)	2579 (37.38%)	698 (37.31%)	1881 (37.41%)	0.96
*APOE* ε4 genotype*				0.01
Non-carrier	5472 (79.32%)	1451 (77.55%)	4021 (79.97%)	
Carrier	1103 (15.99%)	333 (17.80%)	770 (15.31%)	
***Late-life physical activity levels***				
Total MET-minutes/week	3360 (1680, 5600)	3360 (1680, 5400)	3360 (1800, 5760)	< 0.01
Overall physical activity levels (category, %)				< 0.01
Low level	429 (6.22%)	161 (8.61%)	268 (5.33%)	
Moderate level	526 (7.62%)	152 (8.12%)	374 (7.44%)	
High level	5944 (86.16%)	1558 (83.27%)	4386 (87.23%)	
Work-related physical activity (yes, %)	5378 (77.95%)	1380 (73.76%)	3998 (79.51%)	< 0.01
Transport-related physical activity (yes, %)	6225 (90.23%)	1633 (87.28%)	4592 (91.33%)	< 0.01
Recreation-related physical activity (yes,%)	1535 (22.25%)	310 (16.57%)	1225 (24.36%)	< 0.01
Moderate-intensity physical activity (yes,%)	5506 (79.81%)	1403 (74.99%)	4103 (81.60%)	< 0.01
Vigorous-intensity physical activity (yes,%)	672 (9.74%)	149 (7.96%)	523 (10.40%)	< 0.01
***Midlife-to-late-life activity patterns***				
Changes in physical activity levels from midlife to late life (%)				< 0.01
Decreasing activity levels	5790 (83.93%)	1618 (86.48%)	4172 (82.98%)	
Maintaining activity levels	540 (7.83%)	106 (5.67%)	434 (8.63%)	
Increasing activity levels	569 (8.25%)	147 (7.86%)	422 (8.39%)	

*Only includes the 6,575 participants who have *APOE* genotyping data

Continuous variables were described with median and interquartile range [IQR] due to abnormal distribution, and categorical variables with frequency and percentage. Differences between cognitive status groups were assessed using *Wilcoxon* rank-sum test for continuous variables, and *Chi-square* test for categorical variables

### Late-life physical activity levels and cognition impairment

**Fig. 2:**
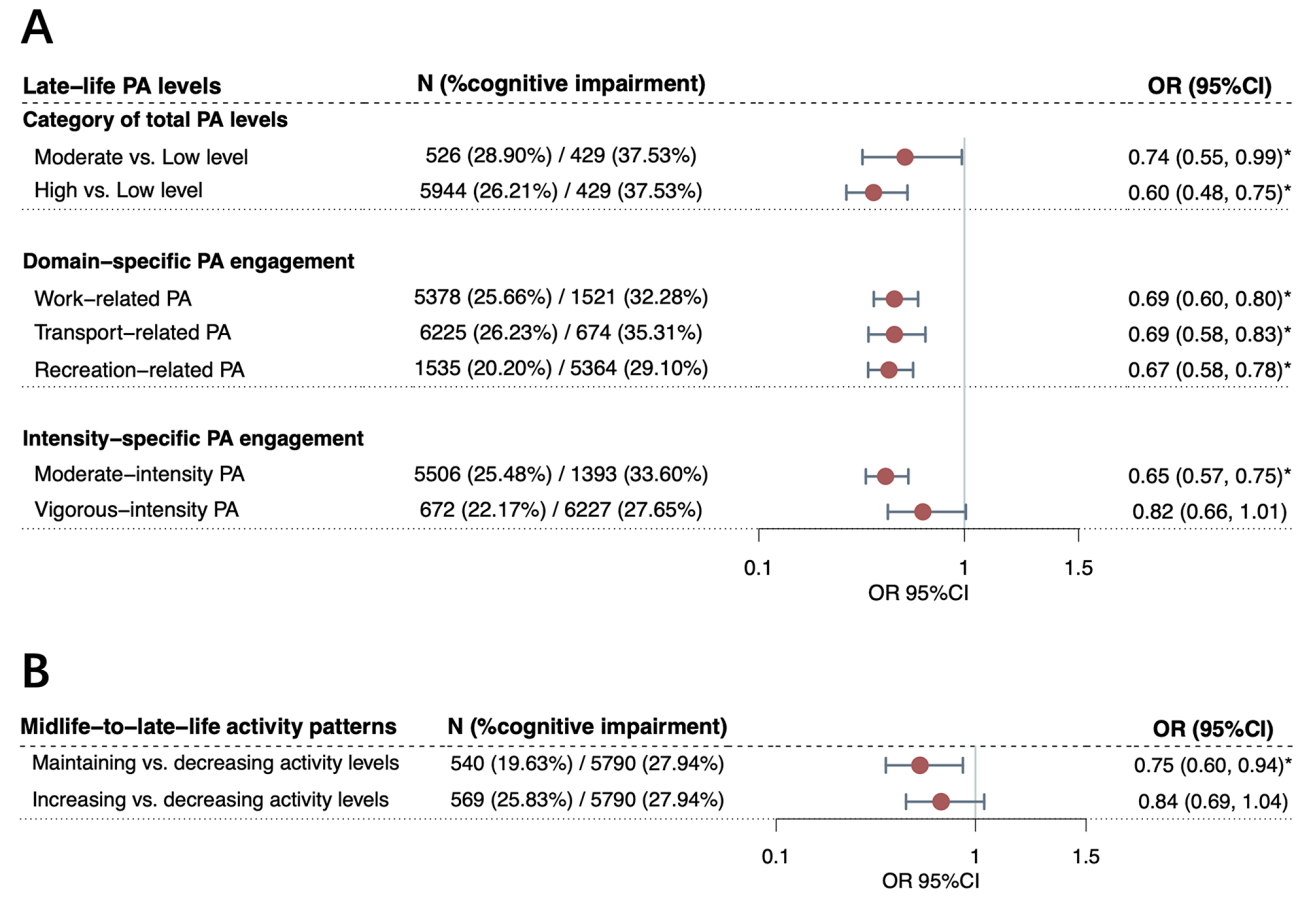
Associations of late-life physical activity and midlife-to-late-life activity patterns with cognitive impairment. Associations between late-life physical activity levels (**A**), midlife-to-late-life activity patterns (**B**), and risk of cognitive impairment. Odds ratios were calculated using multivariate-adjusted models, controlling for age, sex, education attainment, major occupation, body mass index, waist-to-hip ratio, dietary habits, smoking and drinking status, sedentary time, functional limitations, and history of diagnosed hypertension and diabetes. Analyses of domain-specific physical activity, intensity-specific physical activity, and midlife-to-late-life activity patterns were additionally adjusted for overall physical activity levels. Circles represent the point estimates of odds ratios, and error bars represent 95% confidence intervals. One asterisk (*) represents *P*-value < 0.05. PA (physical activity), OR (odds ratio), 95%CI (confidence interval)

Compared to those with low PA levels, participants with moderate (odds ratio [OR] = 0.74, 95% confidence interval [CI] = 0.55 ~ 0.99, *P*-value = 4.16 × 10^− 2^) and high PA levels (OR = 0.60, 95%CI = 0.48 ~ 0.75, *P*-value = 6.35 × 10^− 6^) demonstrated significantly lower risk of cognitive impairment, with an increasing trend from moderate to high PA level group (*P*-trend = 6.57 × 10^− 6^, Fig. 2and Supplementary Table 1). RCS regression demonstrated that such a beneficial link peaked at ~ 4000 MET-minutes/week (equal to ~ 16 h of MPA or ~ 8 h of VPA per week) and then plateaued (Supplementary Fig. 1).

For domain-specific PA, participants engaging in each type of PA (work-, transport-, or recreation-related PA) demonstrated a significantly lower risk of cognitive impairment compared to non-engagers (ORs ranging from 0.67 to 0.69, all *P*-values < 0.05), with recreation-related PA engagement showing the strongest association (OR = 0.67, 95%CI = 0.58 ~ 0.78, *P*-value = 7.88 × 10^− 8^). For intensity-specific PA, engaging in MPA was inversely associated with the risk of cognitive impairment (OR = 0.65, 95%CI = 0.57 ~ 0.75, *P*-value = 4.74 × 10^− 9^) compared to not engaging, with no significant results observed for VPA (Fig. 2and Supplementary Table 1).

Stratified analyses by age and sex revealed largely consistent associations across most subgroups (Supplementary Tables 2–3). The associations for MPA levels lost statistical significance in all stratified analyses but remained directionally consistent, which might be attributed to reduced sample sizes/power in these subgroups (N ranging from 3,007 to 3,892).

### Midlife-to-late-life activity patterns and cognitive impairment

Participants who maintained activity levels from midlife to late life had a significantly lower risk of late-life cognitive impairment compared to those who decreased PA levels (OR = 0.75, 95%CI = 0.60 ~ 0.94, *P*-value = 1.40 × 10^− 2^, Fig. 2and Supplementary Table 1). Age- and sex-stratified analyses showed this association remained significant in the younger group (OR = 0.63, 95%CI = 0.46 ~ 0.87, *P*-value = 4.63 × 10^− 3^) and females (OR = 0.69, 95%CI = 0.51 ~ 0.92, *P*-value = 1.31 × 10^− 2^), but not in the older group and males (Supplementary Tables 2–3). When stratified by overall PA levels, the association between maintaining activity levels and lower cognitive impairment risk persisted in groups with moderate (OR = 0.28, 95%CI = 0.08 ~ 0.98, *P*-value = 4.60 × 10^− 2^) and high PA levels (OR = 0.74, 95%CI = 0.58 ~ 0.95, *P*-value = 1.60 × 10^− 2^, Supplementary Table 4).

### Physical activity, *APOE* ε4 genotype, and cognitive impairment

**Fig. 3:**
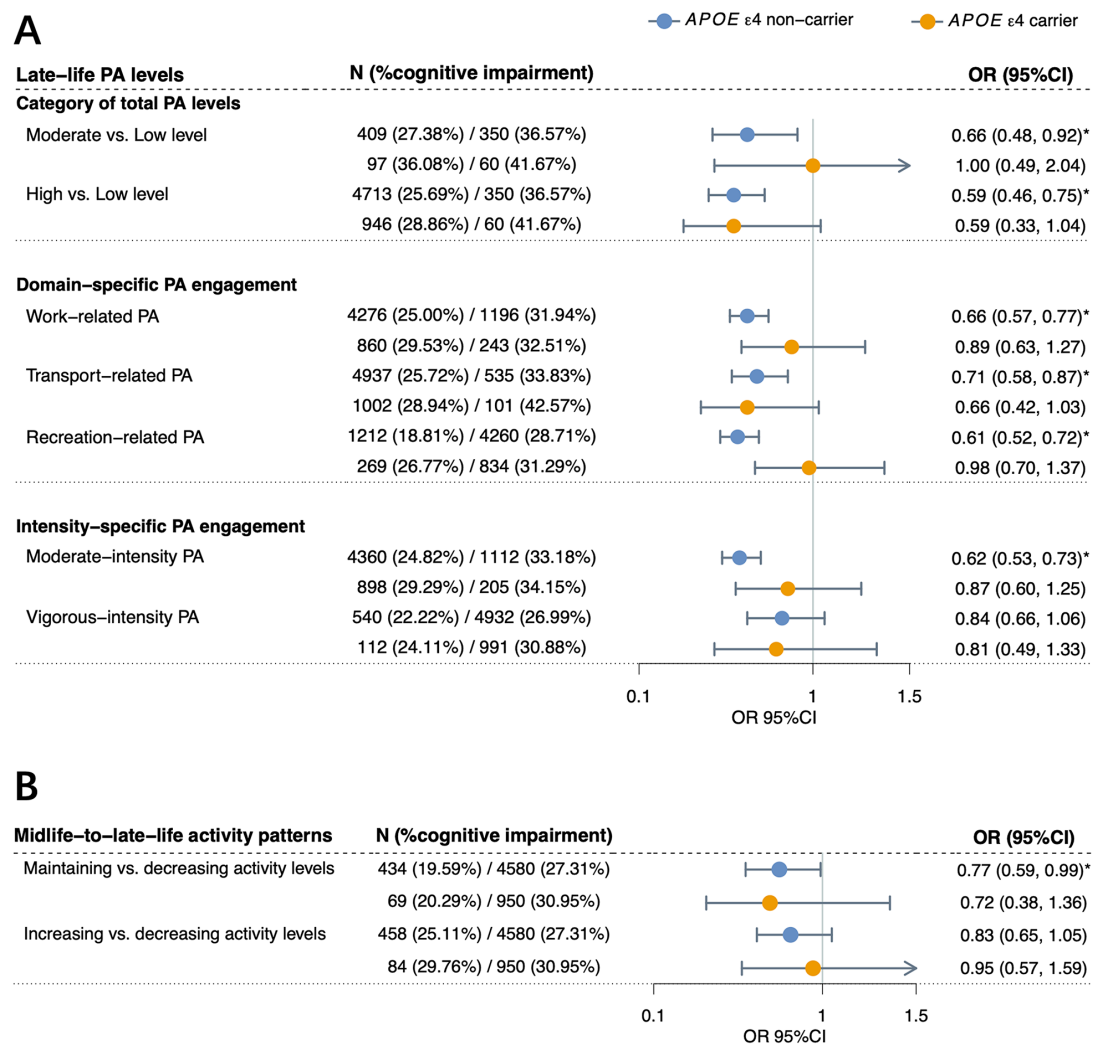
Associations of late-life physical activity and midlife-to-late-life activity patterns with cognitive impairment among participants with different *APOE *ε4 genotype. Associations between late-life physical activity levels (**A**), midlife-to-late-life activity patterns (**B**), and risk of cognitive impairment among participants with different *APOE* ε4 genotype. Odds ratios were calculated using multivariate-adjusted models, controlling for age, sex, education attainment, major occupation, body mass index, waist-to-hip ratio, dietary habits, smoking and drinking status, sedentary time, functional limitations, and history of diagnosed hypertension and diabetes. Analyses of domain-specific physical activity, intensity-specific physical activity, and midlife-to-late-life activity patterns were additionally adjusted for overall physical activity levels. Circles represent the point estimates of odds ratios, and error bars represent 95% confidence intervals. Blue represents estimates in *APOE* ε4 non-carriers, while orange represents estimates in *APOE* ε4 carriers. One asterisk (*) represents *P*-value < 0.05. PA (physical activity), OR (odds ratio), 95%CI (confidence interval), *APOE* (apolipoprotein E)

We observed significant associations in *APOE* ε4 non-carriers, but not in carriers (Fig. 3and Supplementary Table 5). Among *APOE* ε4 non-carriers, moderate (OR = 0.66, 95%CI = 0.48 ~ 0.92, *P*-value = 1.43 × 10^− 2^) and high PA levels (OR = 0.59, 95%CI = 0.46 ~ 0.75, *P*-value = 2.68 × 10^− 5^) were significantly associated with lower risk of cognitive impairment compared to low PA levels. Maintaining activity levels from midlife to late life was also associated with lower cognitive impairment risk in *APOE* ε4 non-carriers (OR = 0.77, 95%CI = 0.59 ~ 0.99, *P*-value = 4.48 × 10^− 2^). A marginal interaction between *APOE* ε4 genotype and recreation-related PA on cognitive impairment risk was identified (*P*-value = 4.48 × 10^− 2^).

**Fig. 4:**
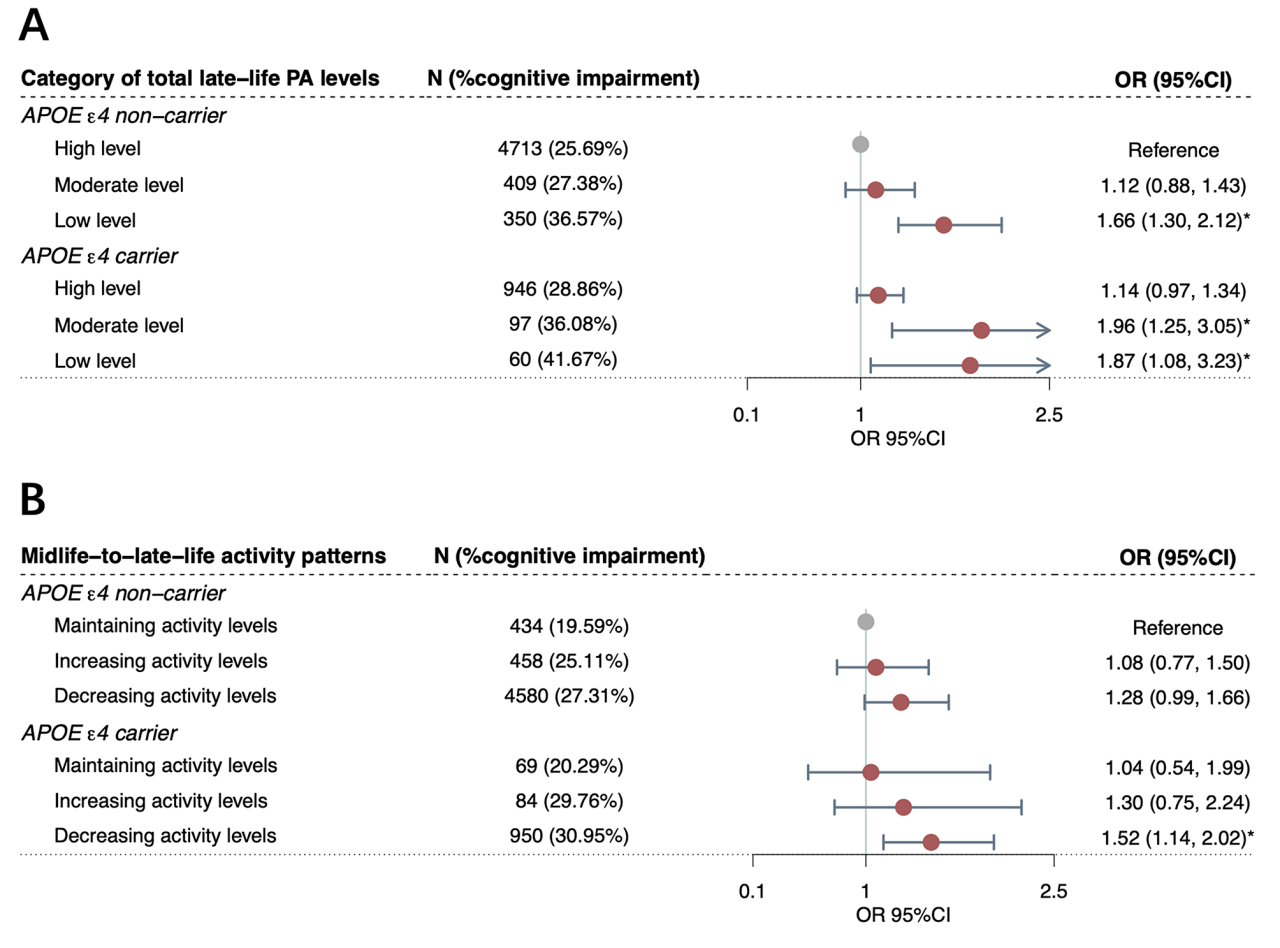
Combined associations of physical activity (late-life physical activity and midlife-to-late-life activity patterns) and *APOE *ε4 genotype with cognitive impairment. Combined associations of physical activity variables (late-life physical activity [**A**] and midlife-to-late-life activity patterns [**B**]) and *APOE* ε4 genotype with risk of cognitive impairment. Odds ratios were calculated using multivariate-adjusted models, controlling for age, sex, education attainment, major occupation, body mass index, waist-to-hip ratio, dietary habits, smoking and drinking status, sedentary time, functional limitations, and history of diagnosed hypertension and diabetes. Analyses of domain-specific physical activity, intensity-specific physical activity, and midlife-to-late-life activity patterns were additionally adjusted for overall physical activity levels. Circles represent the point estimates of odds ratios, and error bars represent 95% confidence intervals. Gray represents the reference groups, while red represents estimates of each combined group compared to the reference group. One asterisk (*) represents *P*-value < 0.05. PA (physical activity), OR (odds ratio), 95%CI (confidence interval), *APOE* (apolipoprotein E)

When *APOE* ε4 genotype and PA variables were jointly analyzed, cognitive impairment risk increased with higher genetic risk and/or lower late-life PA levels (Fig. 4and Supplementary Table 6). Compared to *APOE* ε4 non-carriers with high PA levels, significantly higher cognitive impairment risk were observed in non-carriers with low PA levels (OR = 1.66, 95%CI = 1.30 ~ 2.12, *P*-value = 5.30 × 10^− 5^), carriers with moderate PA levels (OR = 1.96, 95%CI = 1.25 ~ 3.05, *P*-value = 3.10 × 10^− 3^), and carriers with low PA levels (OR = 1.87, 95%CI = 1.08 ~ 3.23, *P*-value = 2.60 × 10^− 2^). Notably, although *APOE* ε4 carriers generally showed higher point estimates of cognitive impairment risk than non-carriers with the same PA categories, we found no significant difference in the risk between carriers with high PA levels and non-carriers with high PA levels (*P*-value > 0.05). Similarly, for midlife-to-late-life activity patterns, cognitive impairment risk did not differ significantly between *APOE* ε4 carriers who maintained activity levels and non-carriers who maintained activity levels (*P*-value > 0.05).

### Sensitivity analyses

Sensitivity analyses yielded largely consistent results with the main analysis (Supplementary Tables 7–17). Late-life PA associations remained consistent across all sensitivity analyses. Midlife-to-late-life activity pattern associations were consistent in most sensitivity analyses, except when using continuous C-MMSE scores as outcomes, where significant results appeared only in the crude model.

## Discussion

This study enhances our understanding of the relationships between PA profiles, *APOE* ε4 genotype, and cognitive impairment in Chinese older adults. Our findings underscore the multifaceted associations of PA with cognitive function, revealing potential beneficial roles of higher overall PA levels, engagement in various PA domains and MPA, and maintenance of activity levels from midlife to late life. Notably, these associations were predominantly observed among *APOE* ε4 non-carriers, indicating they may derive greater cognitive health benefits from optimizing PA profiles. These insights contribute to the development of more targeted approaches to promote cognitive health in aging populations.

Our findings indicate that older adults with moderate and high levels of late-life PA are more likely to have better cognitive health. WHO recommends adults aged 18 ~ 64 and those over 65 should engage in at least 600 MET-minutes/week of PA for basic health benefits, with at least 1,200 MET-minutes/week yielding additional health benefits [1]. Our findings suggest that a higher recommended level of PA for older adults may be particularly beneficial for improving cognitive function, aligning with previous studies reporting the highest PA category best protects against cognitive decline and dementia risk [34, 35]. Notably, our RCS regression analysis revealed a threshold effect for overall late-life PA levels (~ 4,000 MET-minutes/week), beyond which cognitive benefits plateaued, largely corroborating a previous meta-analysis that showed the most pronounced inverse association between PA and cognitive decline before ~ 5,000 MET-minutes/week [6]. These findings collectively suggest that while increasing PA is generally beneficial for cognitive health in older adults, there may be an optimal range for maximizing these benefits.

Our research reaffirms previous findings on the protective effects of recreation-related PA on cognitive outcomes in older adults [9, 36–39], possibly through its physiological impact on improving physical function, strengthening synaptic connections, and increasing cerebral blood flow [40, 41]. We also found protective associations between work-related PA and cognitive impairment, aligning with some studies [9, 37] but contrasting with others that suggest a negative role, known as the “PA paradox” [8]. This discrepancy may be due to differences in population and exposure definition - our participants were primarily retirees likely engaging in moderate household chores and unpaid work, rather than heavy work-related PA. The protective association we observed between transport-related PA and cognitive impairment adds to the limited existing evidence on this topic. Previous studies identified benefits of long-term regular PA, including walking, for cognitive function in 18,766 US older women [42], and cycling for executive function and mental health in 100 older adults [43]. Collectively, findings support a more flexible approach to PA recommendations for older adults - guidance should encourage engagement in various PA domains, including paid or unpaid work, household chores, walking, bicycling, and leisure-time activities. This multifaceted approach may provide older adults with more options to incorporate PA into their daily lives, potentially enhancing adherence and cognitive benefits.

Our study suggests that MPA may be beneficial for cognitive health in older adults, aligning with two studies of 3,722 Japanese and 1,345 US older adults indicating that MPA, but not VPA, was significantly associated with reduced risk of dementia or Alzheimer’s disease [12, 44]. However, a recent study of 91,298 Americans identified a protective association between midlife leisure-time VPA and late-life Alzheimer’s disease-related mortality [45]. This inconsistency might be due to different outcome definitions and PA participation rates, as our low VPA participation (~ 10%) may limit our ability to validate its cognitive health effects. From a public health perspective, the overall decline in health status with aging potentially leads to lower adherence and greater health burdens when engaging in VPA [11, 46, 47]. While higher-intensity activities theoretically provide greater physiological stimulation [48, 49], promoting MPA could be a more practical and feasible strategy for enhancing cognitive health in older adults. Future studies should still focus on the potential benefits of VPA, particularly considering the age and health status of participants.

Our results suggest that maintaining activity levels from midlife to late life may have protective effects against cognitive decline, underscoring the importance of lifelong PA engagement. This finding expands on two previous studies of 3,559 Finnish and 1,345 US older adults which identified associations between maintaining or increasing leisure-time PA from midlife to late life and reduced risk of dementia [38, 44]. Notably, although our results indicated a protective trend for increasing activity levels from midlife to late life, this association did not reach statistical significance, warranting further verification in future studies. Interestingly, the protective association between maintaining activity levels and lower risk of cognitive impairment only persisted in younger groups and females in our stratified analyses, highlighting the potential importance of intervention in the earlier stages of older adulthood, as well as in females. Moreover, our analysis stratified by overall PA levels revealed that the significant associations between maintaining activity levels and lower risk of cognitive impairment persisted in groups with moderate and high PA levels, but not in the low PA level group. This suggests that the cognitive benefits of maintaining activity levels may be contingent on achieving at least a moderate level of PA, emphasizing the importance of not only maintaining but also ensuring an adequate level of PA for cognitive health benefits.

The interactions between PA and *APOE* ε4 genotype on cognitive health remain inconsistent across studies [50], likely due to differences in participant age, sample size, follow-up period, outcome definitions, and PA assessment methods. A study of 7,252 US women revealed stronger associations between midlife VPA and better cognitive trajectories among *APOE* ε4 carriers compared to non-carriers [18]. In contrast, two other studies of 3,559 Finnish and 806 Spanish older adults identified protective associations of an active lifestyle and better cognitive outcomes only in non-carriers [19, 38]. Our findings align with the latter, indicating that optimizing PA profiles may yield more pronounced cognitive benefits in *APOE* ε4 non-carriers. However, it may be due to limited statistical power, as *APOE* ε4 carriers comprised only one-sixth of our participants. Importantly, when combining the *APOE* ε4 genotype and PA variables, we observed no significant difference in cognitive impairment risk between *APOE* ε4 carriers with the highest or maintained activity levels and non-carriers with the same PA groups. This suggests that although the impact of an active lifestyle may be lower among *APOE* ε4 carriers than non-carriers, optimizing PA profiles could still be beneficial regardless of genotype and may, to some extent, offset the increased genetic risk.

Several limitations of this study should be acknowledged. First, the cross-sectional design of the study limits the ability to make causal inferences. Despite the results remaining robust after adjusting for health status factors, such as functional limitations and disease histories, prospective studies are needed to validate our findings and establish temporal relationships. Second, the PA data in this study were based on self-report, which may be subject to measurement and recall biases. Moreover, while GPAQ-v2 is a validated instrument, it neither account for light-intensity PA or activities lasting less than 10 min nor capture certain important dimensions of PA such as whether activities are performed indoors/outdoors, individually/in groups, or their specific focus (e.g., cardiorespiratory vs. strength training). These different aspects of PA might have distinct effects on cognitive health. Future investigations should incorporate more comprehensive or objective measures of PA to validate and extend our findings. Third, since the variables for midlife-to-late-life activity pattern only capture relative changes (decrease, maintain, or increase) rather than absolute changes (such as, they do not distinguish between maintaining low or high PA levels), we also conducted a stratified analysis based on different late-life PA levels, in addition to age and sex, to explore whether the association still existed in groups of the different overall PA levels. Furthermore, some factors such as BMI and WHR could potentially be mediators in the association between PA and cognitive function. Future studies are warranted to explore these potential mediating effects and more complex relationships. Finally, while our main analysis includes a relatively large sample, certain subgroups may still suffer from insufficient statistical power due to smaller sample sizes. These results should be validated using larger populations in future studies.

## Conclusions

This study reveals multifaceted associations between PA profiles and cognitive function in Chinese older adults. Public health strategies should focus on promoting overall late-life PA levels, especially MPA, and maintaining activity levels comparable to midlife, to enhance cognitive health in the aging population. Furthermore, *APOE* ε4 non-carriers may derive greater cognitive health benefits from optimizing their PA profiles. Future prospective studies are needed to confirm these findings.

## Electronic supplementary material

Below is the link to the electronic supplementary material.

Supplementary Material 1

Supplementary Material 2

Supplementary Material 3

Supplementary Material 4

## Data Availability

The datasets generated and/or analysed during the current study are not publicly available due to the participants privacy but are available from the corresponding author on reasonable request.

## Abbreviations

ADL: Activities of daily living
APOE: Apolipoprotein E
BMI: Body mass index
CI: Confidence interval
C-MMSE: Chinese version of mini-mental state examination
GPAQ: Global physical activity questionnaire
IADL: Instrumental activities of daily living
IQR: Interquartile range
MET: Metabolic equivalent task
MPA: Moderate-intensity physical activity
MVPA: Moderate- and vigorous-intensity physical activity
OR: Odds ratio
PA: Physical activity
RCS: Restricted cubic spline
VPA: Vigorous-intensity physical activity
WCHAC: West China health and aging cohort
WHO: World health organization
WHR: Waist-to-hip ratio
