# Supplementary material for: Late-life physical activity, midlife-to-late-life activity patterns, APOE ε4 genotype, and cognitive impairment among Chinese older adults: a population-based observational study
Source: Int J Behav Nutr Phys Act. 2025 Jan 9;22:5. doi: 10.1186/s12966-024-01691-7 (PMC11720804; doi:10.1186/s12966-024-01691-7)
Supplement: Supplementary file 3 — Supplementary Material 3 [file 12966_2024_1691_MOESM3_ESM.docx]

**Supplementary Figure 1. Associations between overall late-life physical activity levels and cognitive impairment using a restricted cubic spline regression model.**

Graphs show odds ratios for cognitive impairment according to overall late-life physical activity levels (measured by total MET-minutes/week) adjusted for age, sex, education attainment, major occupation before or after retirement, body mass index, waist to hip ratio, dietary habits, smoking status, drinking status, sedentary time, functional limitations, and disease histories of hypertension and diabetes. Data were fitted by a logistic regression model, and the model was conducted with 4 knots at the 5th, 35th, 65th, 95th percentiles of overall late-life physical activity levels (reference is 600 MET-minutes/week). Solid lines indicate odds ratios, and shadow shape indicate 95% CIs. OR, odds ratio; CI, confidence interval.
