# Supplementary material for: Late-life physical activity, midlife-to-late-life activity patterns, APOE ε4 genotype, and cognitive impairment among Chinese older adults: a population-based observational study
Source: Int J Behav Nutr Phys Act. 2025 Jan 9;22:5. doi: 10.1186/s12966-024-01691-7 (PMC11720804; doi:10.1186/s12966-024-01691-7)
Supplement: Supplementary file 4 — Supplementary Material 4 [file 12966_2024_1691_MOESM4_ESM.docx]

**The West China Health and Aging Cohort Study**

1. Study Population:

This study utilizes data from the West China Health and Aging Cohort (WCHAC). WCHAC is an ongoing, multi-community prospective cohort study that primarily focuses on adults aged around 60 and above in China. WCHAC aims to investigate social, economic, diet, sleep, and environment risks and multi-omics pathogenic mechanisms related to incidence and progression trajectories of brain related neurodegenerative diseases, bone and joint related degenerative diseases, mental disorders, cardiovascular diseases, metabolic diseases, respiratory diseases in the older population.

1. Recruitment Site:

Participant recruitment was conducted in Hongguang Town, Pidu District, Chengdu City, Sichuan Province. Hongguang Town, a suburban area of Chengdu, is undergoing urbanization, and therefore, this region encompasses both urban and rural representative populations. The Chengdu Pidu District Hongguang Community Health Service Center serves 14 community units, comprising 9 urban units and 5 rural units. The area has a registered permanent population of 26,516 adults aged 60 and above, with a male-to-female ratio of 1:1.16.

1. Study Timeline:

Baseline recruitment occurred from May 2022 to December 2023. Follow-up assessments are planned triennially.

1. Eligibility Criteria:

- Aged around 60 and above
- Provision of informed consent
- Residence in the study area for at least 12 months
- Absence of severe chronic diseases (e.g., stage 3 hypertension) or severe disability
- Adequate communication abilities

1. Baseline Assessment:

The baseline survey included the following modules: health examinations, lifestyle questionnaires, and neuropsychiatric assessments. Biological samples collected included blood, urine, feces, and saliva. The study also collected household dust samples and conducted dynamic monitoring daily physical activity and sleep using accelerometry-based wearable Motion Detectors.

1. Sample Characteristics:

The cohort comprises 10,626 adults aged 54 and above (97.8% aged ≥ 60) from 14 communities, representing 40.17% of the local age-eligible population. The male-to-female ratio is 1:1.23.

1. Follow-up and Data Collection:

For enrolled participants, health records will be collected retrospectively and prospectively through local hospitals, public health monitoring departments, and the Sichuan Provincial Health Commission’s Information Center Disease Diagnosis System. This includes routine health care data, outpatient and inpatient records, and mortality-related information.

1. Multi-omics Data Collection:

The cohort will conduct full or partial biological sample analyses, including:

- Genomic analysis using Illumina’s ASAMD chip (Asian Screening Array Multiple Disease)

Genotype data was imputed to the ChinaMap reference panel. Quality control filters were applied as follows: imputation quality score (INFO) ≤ 0.3 for minor allele frequency (MAF) > 3%, INFO ≤ 0.6 for MAF 1-3%, INFO ≤ 0.8 for MAF 0.5-1%, and INFO ≤ 0.9 for MAF 0.1-0.5%. Additional criteria included a call rate > 97% and Hardy-Weinberg equilibrium (HWE) *P*-value ≤ 1×10^-6^. After these filters, 5,697,579 genetic variants were retained.

- Metabolomics analysis using UPLC-MS/MS (Ultra-Performance Liquid Chromatography-Tandem Mass Spectrometry)
- Brain and cardiac functional MRI scans (Siemens Vida 3.0T MRI)

1. Ethics approval and informed consent

The research protocol was approved by the Medical Ethics Committee of West China Fourth Hospital of Sichuan University (HXSY-EC-2022034). All participants signed informed consent.
